# Pulmonary Hypertension: Pharmacological and Non-Pharmacological Therapies

**DOI:** 10.3390/life14101265

**Published:** 2024-10-04

**Authors:** Jason Tsai, Shaista Malik, Stephanie C. Tjen-A-Looi

**Affiliations:** Susan Samueli Integrative Health Institute, College of Health Sciences, University of California-Irvine, Irvine, CA 92617, USA; smalik@hs.uci.edu

**Keywords:** pulmonary hypertension (PH), pulmonary arterial hypertension (PAH), chronic thromboembolic pulmonary hypertension (CTEPH), cardiac dysfunction, neurostimulation and electroacupuncture, vascular remodeling, alveolar hypoxia

## Abstract

Pulmonary hypertension (PH) is a severe and chronic disease characterized by increased pulmonary vascular resistance and remodeling, often precipitating right-sided heart dysfunction and death. Although the condition is progressive and incurable, current therapies for the disease focus on multiple different drugs and general supportive therapies to manage symptoms and prolong survival, ranging from medications more specific to pulmonary arterial hypertension (PAH) to exercise training. Moreover, there are multiple studies exploring novel experimental drugs and therapies including unique neurostimulation, to help better manage the disease. Here, we provide a narrative review focusing on current PH treatments that target multiple underlying biochemical mechanisms, including imbalances in vasoconstrictor–vasodilator and autonomic nervous system function, inflammation, and bone morphogenic protein (BMP) signaling. We also focus on the potential of novel therapies for managing PH, focusing on multiple types of neurostimulation including acupuncture. Lastly, we also touch upon the disease’s different subgroups, clinical presentations and prognosis, diagnostics, demographics, and cost.

## 1. Introduction

Pulmonary hypertension (PH) is a progressive, incurable clinical condition defined hemodynamically by a mean pulmonary arterial pressure (mPAP) of ≥20 mmHg at rest, as measured by right heart catheterization (RHC) [1,2]. In accordance with the guidelines of the European Society of Cardiology (ESC) and the European Respiratory Society (ERS), PH is generally classified into five etiological groups: pulmonary arterial hypertension (PAH; Group 1), PH associated with left heart disease (PH-LHD; Group 2), PH associated with lung disease or hypoxia (Group 3), PH associated with chronic thromboembolic disease (CTEPH) or other pulmonary artery obstructions (Group 4), and PH with unclear or multifactorial mechanisms (Group 5) [1]. There are multiple subtypes within each group, as listed below in Table 1. In this paper, we focus particularly on the mechanisms and treatments for these groups.

PH patients may also be categorized into different groups based on their functional class (FC); the World Health Organization (WHO), for instance, separates PH patients into groups, from FC I (least severe) to IV (most severe), based on the limitations and symptoms that arise during physical activity (dyspnea, fatigue, chest pain, syncope), as well as comfort at rest [1]. Utilizing the functional classes is important in determining initial treatment plans and defining goals in stages, for both the patient and physician. For instance, physicians can set the goal as containing symptoms at FC I or II during the management of PH [1,3]. As such, the survival rate is better in patients who improve from a more severe to a less severe FC, compared to those remaining at the same FC [4,5].

This review article will explore how care teams treat PH, including exploring FDA (Food and Drug Administration)-approved and potential emerging therapies for the disease as well as the mechanisms they target. The paper will also mention the diagnosis, cost, and demographics of the disease.

## 2. Demographics and Cost

The prevalence of PH varies significantly across different populations. Older patients over the age of sixty-five have an estimated PH prevalence of 10%, and those with comorbidities such as aortic stenosis or heart failure with preserved ejection fraction (HFpEF) have a higher prevalence of PH, ranging from around 30 to 90% [6,7,8,9]. Despite scanty evidence, studies also suggest that those with residence in economically developing nations, poor healthcare access, low annual income, higher risk of diseases such as tuberculosis and human immunodeficiency virus (HIV) infection, and low health literacy have a higher prevalence of PH and worse FC and are less likely to receive adequate diagnosis and treatment [10,11,12,13,14,15]. Furthermore, multiple studies find that PH-LHD is the most common type of PH, followed by PH due to lung disease and/or hypoxia [1,6].

Through mechanisms such as inflammation and vascular injury, COVID-19 (Coronavirus Disease 2019) cohorts are also associated with a higher prevalence of PH. This could be in part due to the higher prevalence of pre-existing lung and heart comorbidities in patients affected by COVID-19; consequently, such patients could have already developed PH previously [16,17,18]. On the other hand, some studies suggest that COVID-19 could precipitate elevated pulmonary arterial pressure and PH in patients, from those with venous thromboembolic disease to those without any previous cardiovascular complications [19,20,21,22]. Indeed, multiple studies have reported that COVID-19 could contribute to acute or chronic cardiovascular and pulmonary events in patients that did not previously have them, including increased pulmonary arterial pressure, diastolic dysfunction, pericardial thickening, and impaired left ventricular function [23,24,25]. Regardless, given the lack of abundant data, particularly those which establish patients as having no prior signs of PH, more studies are necessary to establish PH as a COVID-19 sequela.

Reports also indicate that the prevalence of PH is rising, in part due to the increasing use of diagnostic tools such as echocardiography, an aging population, and the increasing burden of diseases such as heart failure [26,27]. Consequently, it is necessary to understand and tackle certain risk factors associated with PH, such as disease prevention and healthcare equity programs, along with finding novel therapies [1,10].

PH can also impose a large cost on the patient, their caregivers, and the broader economy. For instance, in the United States (US), drugs for PAH can cost anywhere between USD 5000 and USD 250,000 annually according to different studies, which is evidently compounded for those on combination therapies or in worse FCs; similar costs for PAH-related drugs have also been reported in other countries [28,29,30,31,32]. Including costs such as hospitalizations, medical devices, diagnostics, supportive therapies, and transplants, PAH can cost patients upwards of tens of thousands of dollars and countries hundreds of millions of dollars [28,29,30,33]. For other types of PH, such as PH associated with systemic sclerosis and PH associated with chronic obstructive pulmonary disease (COPD), total costs per patient may also amount to thousands of dollars annually [34,35,36]. Moreover, studies on patients with PAH and CTEPH report that they have their work and consequently their income significantly affected by the disease, due to factors including being unable to work at all, taking extended sick and disability leave, or considering early retirement. Similarly, caregivers of these patients often have their own work and income affected and experience exhaustion, leading to an indirect annual cost of thousands of dollars and tens of millions of dollars nationally [28,33,37,38,39,40].

Those with PH also often present with comorbidities that increase the cost. For instance, renal disease is associated with PH: studies estimate that PH affects 17 to 56 percent of patients receiving hemodialysis, with a varying incidence between 9 and 39 percent in those with end-stage renal disease (ESRD) [41,42,43,44]. Thyroid disease has also been associated with PH, with one study reporting the prevalence of PH to be 35–47% in patients with hyperthyroidism and 10–24% in patients with hypothyroidism; interestingly, reports suggest a mechanistic link between thyroid hormones and the pulmonary vasculature [45,46]. Furthermore, as mentioned previously, both PH-LHD and PH associated with chronic lung disease and/or hypoxia are the most common groups of PH. The cost of managing these diseases in combination with PH could place additional burden on both the patient and their caregivers.

## 3. Diagnosis and Biomarkers

Diagnosis and classification of PH relies on mPAP; indeed, medications such as riociguat, epoprostenol, and sildenafil decrease mPAP in patients and provide symptomatic benefit [47,48]. However, additional measures, such as pulmonary arterial wedge pressure (PAWP; mmHg units) to measure pulmonary vein and left-sided heart pressure and pulmonary vascular resistance (PVR; Wood units) to measure pulmonary arterial resistance and narrowing, are important in the classification of PH [1].

**Figure 1:**
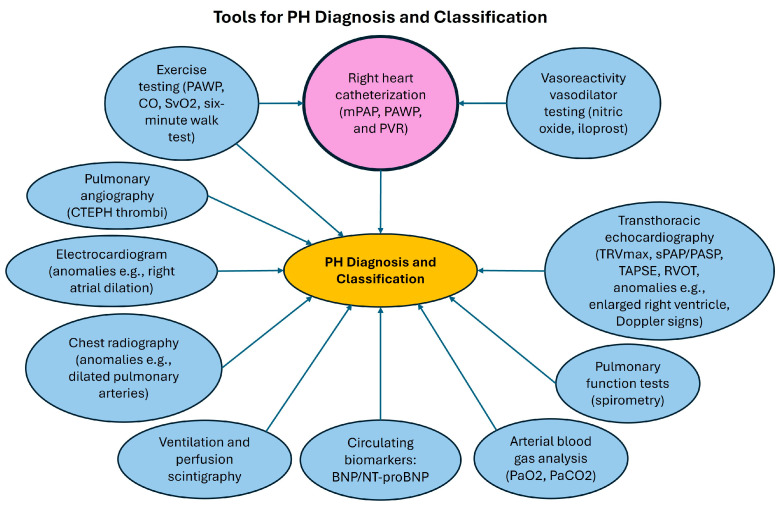
Tools used for pulmonary hypertension (PH) diagnosis and classification. Right heart catheterization is uniquely colored because it is required for the diagnosis of PH. Tools such as exercise testing or vasoreactivity testing can be utilized when performing right heart catheterization. This figure is only a summary: it does not include all the parameters that can be measured through diagnostic tools (e.g., chest radiography can also measure factors such as right atrial dilation) nor novel diagnostics currently being explored but not yet established (e.g., serum uric acid). mPAP = mean pulmonary arterial pressure, PAWP = pulmonary arterial wedge pressure, PVR = pulmonary vascular resistance, CO = cardiac output, SvO_2_ = mixed venous oxygen saturation, CTEPH = chronic thrombo-embolic PH, BNP = brain natriuretic peptide, NT-proBNP = N-terminal prohormone of BNP, TRVmax = maximum peak tricuspid regurgitation velocity, sPAP/PASP = pulmonary arterial systolic pressure, TAPSE = tricuspid annular plane systolic excursion, RVOT = right ventricular outflow tract, PaO_2_/PaCO_2_ = partial pressure of oxygen/carbon dioxide.

Multiple diagnostic techniques can be utilized when diagnosing and classifying PH through various parameters (Figure 1); however, official ESC/ERS guidelines require RHC for diagnosing and classifying PH. Care teams typically provide RHC as an outpatient procedure in cardiac catheterization labs at experienced centers, whereby a catheter is inserted into a patient’s arm, neck, groin, or other areas to measure pressures inside the heart and lungs [1]. The test measures parameters including mPAP, PAWP, and PVR for subsequent classification into pre-capillary PH (often due to pathological pulmonary vascular remodeling and resistance), post-capillary PH (often due to conditions such as left-sided heart failure), or combined pre- and post-capillary PH; a PAWP of ≤15 mmHg is generally necessary for a diagnosis of pre-capillary PH, including PAH [1,2]. Differentiating between these groups is crucial to avoid misclassification and mistreatment in PH patients [1].

Care teams often perform additional tests, such as vasoreactivity and exercise testing, and measure further parameters, such as mixed venous oxygen saturation (SvO_2_) and cardiac output (CO), while conducting RHC [1]. For instance, in vasoreactivity challenges, patients with PAH inhale compounds such as nitric oxide (NO) or iloprost to evaluate their pulmonary arteries’ response to vasodilators and their candidacy for calcium channel blocker therapies; in this test, CO increasing or remaining unchanged helps indicate a positive response [1]. Furthermore, care teams can use RHC with exercise testing to assess patients with symptoms such as unexplained dyspnea during physical activity; in such tests, teams measure parameters such as PAWP and CO help diagnose exercise PH, while guidelines also recommend taking SvO_2_ and arterial saturation [1].

In addition to RHC, clinicians often use echocardiography, particularly transthoracic echocardiography (TTE), as a first-line tool when there is suspicion of PH, because it is non-invasive and provides valuable information. Official ESC/ERS guidelines recommend measuring the maximum peak tricuspid regurgitation velocity (TRVmax), which confers a high probability for PH if it is >2.8 m/s [1]. Another important parameter is pulmonary arterial systolic pressure (sPAP, PASP) [1,49]. Although there are potential inaccuracies in measuring variables that contribute to sPAP, such as right atrial pressure, an sPAP above 60 mmHg can also confer a high probability of PH [49]. Other important echocardiographic parameters include tricuspid annular plane systolic excursion (TAPSE) and right ventricular outflow tract (RVOT). Such values are not only important in suggesting disease—indeed, lower TAPSE/sPAP ratios may support a diagnosis of PH—but also can help differentiate between subgroups of PH—for instance, different RVOT patterns may indicate pre-capillary PH or PH with HFpEF [1,49,50,51].

TTE can also analyze other signs which point to PH, such as enlarged right ventricles, a flattened interventricular septum, or left ventricular hypertrophy [1,49]. Such signs can also be important in the classification of PH; for instance, in PH associated with HFpEF, left ventricular hypertrophy and a deviated interatrial septum tend to be present [49]. Similarly, left atrial enlargement can indicate PH-LHD [49]. Other signs that clinicians can measure include Doppler echocardiographic signs, such as E/A (early diastolic mitral inflow velocity to late atrial contraction mitral inflow velocity) or E/E′ (early diastolic mitral inflow velocity to early diastolic mitral annulus velocity) ratios. Lastly, it is important to note that while TTE can provide a probability estimate for PH, RHC is still necessary for a definitive diagnosis.

Multiple other tests may also aid in diagnosis. For instance, electrocardiograms can provide evidence for right atrial dilation, right ventricular hypertrophy and strain, right axis deviation, P pulmonale, right bundle branch block, and a prolonged QTc interval (corrected interval between Q and T waves) [1,48,52,53]. Because patients with PH can face complications such as arrythmias, electrocardiograms can be crucial in establishing treatment, such as rhythm-control therapy [1,53,54]. Chest radiography can also check for comparable results to echocardiography, including right atrial enlargement, dilated pulmonary arteries, calcifications, and aneurysms [1,48,55]. Pulmonary function tests and arterial blood gas analysis can help distinguish between groups such as idiopathic PAH and PH associated with lung disease by measuring parameters such as total lung capacity and forced expiratory volume, while ventilation/perfusion scintigraphy and pulmonary angiography can help diagnose CTEPH [1]. Other tests include exercise tests and abdominal ultrasound for portal hypertension [1]. Typically, PAH diagnosis occurs only after excluding other groups of PH as defined in Table 1 [1,56].

In addition to tests, different biomarkers can be crucial in assessing PH early. One such biomarker is brain natriuretic peptide (BNP) and its inactive, more stable counterpart, N-terminal prohormone of BNP (NT-proBNP). Both peptides derive from proBNP (prohormone of BNP) in a process which occurs in ventricular cardiac myocytes in response to stretch, such as stretch due to increased pressure or volume overload. Consequently, a blood test can also measure both peptides to aid in the diagnosis of PH [1,57]. Indeed, elevated BNP is present in idiopathic PAH, PH associated with COPD, CTEPH, and multiple other classes of PH; similarly, one study reports elevated NT-proBNP in a heterogeneous group of patients with pre-capillary PH [57]. Moreover, along with other markers, such as functional class, as mentioned above, and the six-minute walk test, both BNP and NT-proBNP are strong predictors of mortality in PAH patients and, in part, constitute PAH risk stratification tools [1,57]. Currently, BNP and NT-proBNP are the primary biomarkers measured at PH centers to evaluate for the disease [1]. However, it is important to note that BNP and NT-proBNP are not specific for PH and can increase in other cardiac and renal diseases [1,57].

In addition to BNP/NT-proBNP, multiple markers could aid as potential diagnostic or predictive markers in PH, although care teams have yet to apply them clinically as such. For instance, increased serum endothelin-1 (ET-1), a potent vasoconstrictor, and reduced serum NO, an important vasodilator, are both implicated in PH, and these compounds are mentioned in more detail below [57]. Hyperuricemia is also present in patients with PAH and PH-LHD, and studies report that serum uric acid levels correlate with mortality in PAH patients; however, hyperuricemia is also present in other diseases and other states, such as renal dysfunction or in cases of diuretic use [57]. Additional studies are investigating factors such as bone morphogenic proteins (BMPs) and chemokines as biomarkers for PH [57].

Those with PH typically present with symptoms not entirely specific to the disease but important for physicians to suspect and initiate prompt action for therapy and referral. For instance, a cardinal feature of PH is progressive exercise dyspnea, while other symptoms are fatigue, tachycardia, and syncope upon exertion. On physical examination, patients may also display lower extremity edema, dilated jugular veins, and ascites. Additional symptoms observed in PH patients are augmented heart sounds, such as a pronounced pulmonary component in the second heart sound, cyanosis, and exertional and nocturnal hypoxia. Moreover, mental stress due to sleep problems, anxiety, and depression can arise in patients with PH, in which case treatment plans could include adequate psychosocial support and the appropriate medications [1,58,59].

## 4. Mechanisms and Established Treatments

PH is associated with multiple changes at a tissue and cellular level. The vasoconstriction of the pulmonary arteries with the potential involvement of the pulmonary vein and capillaries contributes to an elevated mPAP [1]. Histologically, patients with PAH and CTEPH show various forms of pathological vascular remodeling, vascular lesions, and associated inflammation [48,60,61]. Multiple studies also report pulmonary vascular pathology, such as arterial thickening and hypertrophy, in those with PAH, CTEPH, and PH-LHD [62,63,64,65]. Vascular fibroblast migration, increased production of matrix proteins, endothelial cell proliferation, and neovascularization could also contribute to PH pathology [61,62,66,67]. Lesions also increase the chance of surgical risk in patients with CTEPH [48,68,69].

Hypoxia, infection and inflammation, shear stress, genetics, vasodilator–vasoconstrictor imbalances, and dysautonomia contribute to or are results of such vascular pathology in PH. The sections below will discuss these mechanisms and related treatments.

### 4.1. Vasoconstrictor–Vasodilator Imbalances

PH has long been associated with the increased production of vasoconstrictors, such as endothelin-1 (ET-1), and the decreased production of vasodilators, such as NO and prostacyclin.

For instance, ET-1 is overexpressed in both the plasma and lungs of PH patients in multiple different PH groups, such as CTEPH, PH associated with hypoxia, and PAH; one study also reports that ET-1 concentration positively correlates with the severity of symptoms in CTEPH patients [70,71,72,73,74]. Mechanistically, ET-1 activates two G-coupled protein receptor subtypes, endothelin receptor A (ET_A_) and endothelin receptor B (ET_B_). ET_A_ receptors typically dominate over ET_B_ receptors in the pulmonary vasculature; the activation of the former leads to vasoconstriction while the latter induces vasodilator release [73,75,76]. ET-1 promotes intracellular calcium release through the phospholipase C (PLC)/inositol triphosphate pathway, causing smooth muscle contraction. Diacylglycerols produced by PLC activation further potentiate smooth muscle contraction [76,77]. Furthermore, ET-1 can induce the transcription of genes to promote smooth muscle cell growth and proliferation by the extracellular signal-regulated kinase (ERK) or sodium/hydrogen pump [75,78,79,80].

In accordance with these mechanisms, multiple current drugs aim to block ET_A_ activation to help manage symptoms; however, these drugs are more specific to PAH and have minimal effect on improving symptoms such as exercise capacity in other groups of PH patients [1,81,82,83,84]. Endothelin antagonists include drugs such as bosentan, ambrisentan, and macitentan. Bosentan and macitentan are both oral medications that competitively inhibit ET_A_ and ET_B_ receptors, promoting pulmonary vasodilation through the pathways mentioned above [1]. Ambrisentan is also an oral medication but selectively inhibits ET_A_ receptors. Although there are side effects associated with these medications, including liver dysfunction and thrombocytopenia, these drugs are effective in improving mortality and morbidity rates, WHO-defined FC, and exercise capacity as measured by the six-minute walk distance test, as well as hemodynamic parameters such as PVR and mPAP in patients with PAH [85,86,87,88].

Vasodilator mechanisms also play a vital role in the pathogenesis of PH. For instance, ET_B_ receptor activation on endothelial cells can trigger nitric oxide synthase (NOS) to produce NO; NO subsequently diffuses to vascular smooth muscle cells and activates soluble guanylate cyclase (sGC), which generates cyclic guanosine monophosphate (cGMP) that leads to smooth muscle relaxation and vasodilation [78]. Moreover, NO synthesis depends on multiple additional factors: for instance, NOS often uses both oxygen and L-arginine to produce NO [89]. Additionally, studies not only report reduced lung expression of NOS in those with PAH and secondary PH, but also increased phosphodiesterase 5 (PDE5) expression, an enzyme which degrades cGMP, in PAH lung tissue [90,91].

There are multiple drugs which target these pathways; however, these drugs are more specific for PAH rather than PH-LHD or PH associated with chronic lung disease or hypoxia. For instance, PDE5 inhibitors (PDE5i) include tadalafil and sildenafil, which target the NO pathway by acting as reversible competitive inhibitors of PDE5; they both improve hemodynamic values and functional status in patients with PAH [92,93]. There are also sGC stimulators including riociguat, which help to treat those affected by both PAH and CTEPH. For instance, riociguat targets the NO pathway by increasing the sensitivity of sGC to NO and directly stimulating the sGC enzyme independently of NO. Accordingly, riociguat also helps increase exercise, improve FC, and improve hemodynamic parameters in these patients [47,94].

Prostacyclin is a small lipid molecule that acts on prostacyclin (IP) receptors on the smooth muscle to induce potent cyclic adenosine monophosphate (cAMP)-mediated vasodilation and anti-proliferative pathways [1,95]. However, research finds that PAH patients can have abnormalities in the prostacyclin pathway, such as a reduction in lung and pulmonary arterial prostacyclin synthase expression [1,95]. Similar to drugs targeting the NO pathway, prostacyclin analogs aim to correct vasoconstrictor–vasodilator imbalances by binding to prostacyclin receptors present on endothelial cells, smooth muscle cells, and platelets. In binding to these receptors, they promote cAMP production in both smooth muscle cells and platelets, promoting vasodilation and inhibiting platelet aggregation [1].

Prostacyclin analogs for PAH treatment include epoprostenol, iloprost, and treprostinil. Clinicians can administer epoprostenol intravenously, and the drug is more effective for those in more severe FC compared to other therapies [96,97]. Although there are more stable versions, epoprostenol typically has a short half-life and consequently requires continuous infusion [1]. Regardless, intravenous (IV) epoprostenol improves symptoms such as exercise capacity as well as hemodynamic parameters [96].

Treprostinil is similar to epoprostenol but allows administration via subcutaneous injection or inhalation; through these means, it has shown tremendous benefit in improving exercise capacity, hemodynamic parameters, symptoms, quality of life measures, and even certain biomarkers such as NT-proBNP [98,99]. Oral treprostinil shows more mixed results in benefits, as measured by the 6 min walk test [100,101]. Interestingly, inhaled treprostinil has shown marked benefits in PH patients with interstitial lung disease; in the INCREASE clinical trial, patients undergoing the treatment for 16 weeks showed better exercise capacity, an increase in time until certain clinical worsening events (such as hospitalization, transplantation, or death), and a reduction in NT-proBNP. Despite limitations such as in measuring what constituted an acute exacerbation, these studies report treprostinil effectively helps a subset of patients with Group 3 PH [102,103].

Iloprost is another inhaled prostacyclin analog that has shown benefit symptomatically and hemodynamically in PAH patients [104]. Selexipag is yet another prostacyclin receptor agonist and can be orally available; clinical trials have shown that it helps to ameliorate hemodynamic parameters and reduce the relative risk of morbidity and mortality events [105,106]. Lastly, beraprost improves exercise capacity, at least in the short term, for PAH patients, but not hemodynamic parameters [107,108].

For PAH patients, a combination of a PDE5i and endothelin receptor antagonist is often the standard of care for both mild and more severe cases [3]. Typically, common side effects of prostacyclin analogs vary, but include headache, flushing, jaw pain, diarrhea, and errors related to administration, such as IV injection site infection, infusion site pain, and sepsis [1].

### 4.2. Hypoxia and Oxygen Therapy

Hypoxia is known to be a contributing factor to PH in both patients and animal models; the mechanisms underlying this process, however, are multifactorial and not well-established in PH. Generally, pre-clinical studies have discovered that low oxygen can drive hypoxic pulmonary vasoconstriction (HPV) in smooth muscle cells; research has suggested that these cells are responsible for sensing changes in oxygen, but the nature of this oxygen sensor is less clear [109,110]. Moreover, the nature of HPV in PH is unclear, given that studies on patients who lived, in part, in high altitudes still maintain a relatively elevated pulmonary arterial hypertension even when entering normoxic conditions [109].

Studies on animal pulmonary arterial tissue and vascular smooth muscle cells report that hypoxia can trigger alterations in mitochondria-mediated reactive oxygen species (ROS) production, ROS-dependent reduction in NO bioavailability and NO-related enzyme activity, and redox-dependent modification of potassium and calcium channels in smooth muscle cells, promoting vasoconstriction [111,112,113,114,115,116,117,118]. Indeed, a blood-based study on those living in high altitudes showed that they have an increased pulmonary output of free radicals, inflammatory cytokines, and a loss of NO bioavailability, suggesting PH and HPV could be related via ROS and a loss of available pulmonary NO [119]. Moreover, patients with PAH display elevated levels of arginase, an enzyme that competes with NOS for the substrate arginine; similarly, pre-clinical research also shows that hypoxia can reduce arginine transport into endothelial cells for NO synthesis [120,121].

There is also evidence that pulmonary neuroendocrine cells (PNECs), oxygen-sensitive chemoreceptors in the alveolar and bronchiole endothelium, may play a role in the pathogenesis of PH. For instance, studies on lung tissue show an increased amount of PNECs in patients with PAH, as well as related diseases such as COPD [122,123]. Moreover, PNECs store important vasoconstrictors such as serotonin and vasodilators such as calcitonin gene-related peptide (CGRP) [123,124]. Given that animal studies have shown that hypoxia can trigger serotonin release from and CGRP depletion in PNECs [123,125], it is possible that PNECs can play a crucial role in hypoxia-associated PH and HPV; however, researchers must conduct more studies on PNECs to establish their link to PH.

Hypoxia also activates multiple hypoxia-inducible factors (HIFs) [126,127]. In animal and human cellular models, both low oxygen and ROS, triggered by hypoxia, play a role in promoting hypoxia-inducible factor 1 (HIF-1) stabilization and signaling [128,129,130,131]. HIF-1 is also associated with vascular remodeling and smooth muscle hypertrophy in hypoxic animal models [132] as well as multiple molecular changes, including the downregulation of potassium channel expression and the upregulation of sodium–hydrogen exchangers in animal smooth muscle cells, both of which are associated with smooth muscle hypertrophy and hyperplasia [133,134]. Hypoxia also increases ET-1 expression through HIF-1 complex-mediated binding to the transcription site for *ET-1* in human cell models [135]. Moreover, in endothelial cells from patients with PAH and CTEPH, HIF-1 subunits are overexpressed [136]; endothelial cell research from CTEPH patients also suggests that HIF-1 can play a role in controlling platelet adhesion to endothelial cell walls through activating the neural precursor cell expressed developmentally down-regulated protein 9 (NEDD9) protein [136,137]. Lastly, human and animal cell studies show that hypoxia triggers fibroblast migration and proliferation through complex signaling pathways involving protein kinase B (Akt), as well as vascular remodeling through HIF-mediated increases in activin, a ligand associated with PH [138,139,140].

With regard to treatment, care teams can supplement oxygen therapy to maintain pulmonary and systemic oxygen levels when pO_2_ is low in each group of PH [1]. Clinical studies on PAH have reported that oxygen therapy does not have a significant clinical or survival effect on the prognosis [1,141]. However, other clinical studies have also shown oxygen helps patients with PAH, including by lowering hemodynamic values and providing symptomatic benefit such as exercise function [110]. In this respect, it is possible that oxygen therapy helps those with PH via the mechanisms listed above, specifically by promoting vasodilation and reducing HPV. Regardless, ESC/ERS guidelines recommend reserving oxygen for those with low O_2_ saturation during exercise or sleep, although care teams can employ ambulatory oxygen if there is symptomatic benefit [1].

### 4.3. Genetics and Signaling Pathways

Imbalances in proliferative and anti-proliferative signaling via the transforming growth factor-β (TGF-β) receptor have been associated with PAH. For instance, mutations in the BMP receptor type II (*BMPR2*) gene, a subtype of TGF-β receptor, are the most common heritable form of PAH and are associated with increased intima, smooth muscle, and adventitia thickening in pulmonary arteries through complex signaling pathways [61]. However, it is likely that mutations in other genes constitute the pathology of PAH, since not all of those with *BMPR2* gene mutations develop the PAH phenotype.

BMPR2 signaling is complex and controls multiple signaling pathways. First, BMPR2 downstream targets include BMP4 (bone morphogenic protein 2) and BMP7 (bone morphogenic protein 7) in human vascular smooth muscle cells, which help repress the secretion of ET-1; moreover, ET-1 can downregulate BMPR2 expression and signaling in these cells [142,143]. This could underlie, in part, the reason those with *BMPR2* gene mutations are at risk for developing PAH.

Immunologically, BMPR2 deficiency is also associated with increased cytokine production from human vascular smooth muscle cells, which is associated with reductions in extracellular superoxide dismutase 3 (SOD3) activity and the increased production of ROS [144]. Additionally, pre-clinical studies on both animal and human cell and tissue models, including those modeling PAH, associate BMPR2 deficiency with increased lymphocyte, neutrophil, monocyte, and macrophage activity and migration [145,146,147]. This is understandable, given that BMPR2 activation on macrophages can inhibit macrophage activation and the upregulation of adhesive membrane proteins [145,146,147]. Interestingly, from a viral standpoint, HIV infection can lead to the miRNA-mediated downregulation of BMPR2 expression [148].

Metabolically, BMPR2 loss is associated with mitochondrial dysfunction in endothelial cells and cardiomyocytes by shifting away from glucose and fatty acid oxidation, respectively; studies suggest that these changes can predispose PAH clinical symptoms such as endothelial inflammation and right ventricular failure [149,150].

More pertinently, impaired BMPR2 activity may result in the impaired regulation of activin, a pro-proliferative ligand that reduces bone morphogenic protein (BMP) activity and promotes myogenic proliferation and remodeling; indeed, activin A is overexpressed in pulmonary vessels of both human and animals affected by PAH [151,152,153]. Accordingly, sotatercept, a fusion protein which acts as a ligand trap to inhibit activin signaling, improves clinical outcomes for PAH patients, including exercise capacity, as measured by the six-minute walk test [154,155]. By inhibiting activin’s activity, sotatercept also interferes in activin’s ability to compete with surface receptors with anti-proliferative BMP signals.

### 4.4. Inflammation

Inflammation is a crucial factor in the pathology of PH, especially in those with PH associated with infection or connective tissue diseases. For instance, vascular lesions in human lung samples harbor lymphocytes, dendritic cells, mast cells, macrophages, and proliferating endothelial cells [156,157,158,159]. Patients with PH also show higher serum cytokines including multiple interleukins (IL), tumor necrosis factor alpha (TNFα), and chemokines [160,161,162].

Multiple cytokines and pathways contribute to PH pathogenesis. For instance, one study on a mice model showed that hypoxia increases lung production of IL-1β, which activates receptors on smooth muscle cell and triggers proliferation [163]. Moreover, IL-6 triggers the signal transducer and activator of transcription 3 (STAT3) in human cell cultures, causing the increase in miRNAs that degrade BMPR2 mRNA; as mentioned previously, a loss of BMPR2 signaling can contribute to PH pathology [164]. Indeed, studies on animal models and human cell cultures have shown that Il-6 can increase endothelial growth factor receptors and matrix metalloproteinases that promote smooth muscle cell proliferation and the endothelial–mesenchymal transition seen in PH [165,166]. Additionally, research suggests that IL-6 and other cytokines, such as IL-8 and IL-10, can be important predictors for survival in PAH patients, similarly to traditional markers such as the 6 min walk test or hemodynamic parameters, signifying their importance in PH pathogenesis [160,167]. Other interleukins, such as IL-8 and IL-13, act through pathways that promote endothelial cell proliferation and arginase upregulation, respectively [168,169].

The mechanisms related to increased lymphocyte activity and consequent cytokine production are less clear in PH. Studies have reported that patients may have dysregulated regulatory T-cell function which could predispose high immune system reactivity [170,171]. A considerable proportion of PH patients have circulating autoantibodies [172,173], which could precipitate endothelial cell damage and further PH pathogenesis. Infections such as HIV or COVID-19 are also associated with damage to the vascular endothelia, cytokine storms, increased inflammation, and PH pathogenesis. Research studies report that COVID-19 potentiates intense platelet activation, which might contribute to thrombosis seen in multiple groups of PH patients [174,175].

With regard to therapies, PAH-specific drugs have limited anti-inflammatory effects, and prostacyclins are even stronger mediators of immune activity [176]. For instance, sotatercept not only acts as an activin ligand trap and restores BMP signaling, but also contributes to immunosuppression: in a rat model of PAH, sotatercept reduced pulmonary inflammation and inflammatory gene expression profiles [177]. Another example is riociguat, which in part mediates inflammation by blocking TGF-β signaling and inhibiting fibrosis, as shown in a pre-clinical study [178]. In animal models, bosentan suppresses the cytokine TNFα; in patients with PAH due to systemic sclerosis, bosentan leads to a reduction in proinflammatory cytokines [179,180].

### 4.5. General Therapies

ESC/ERC guidelines do not recommend most PAH-specific drugs for those with PH-LHD or PH associated with chronic lung disease or hypoxia, as the evidence suggests a lack of clinical improvement and potentially adverse outcomes [1]. However, the FDA has approved riociguat for helping those with CTEPH, and reports suggest that macitentan can also benefit CTEPH patients [1,181]. Moreover, diuretics are a general supportive therapy for PH patients to relieve pulmonary congestion and right-sided heart failure [1].

For patients with CTEPH, pulmonary thromboendarterectomy (PEA) is typically the treatment of choice and is potentially curative; physicians can also employ percutaneous balloon pulmonary angioplasty (BPA) if PEA is ineffective or unfeasible due to surgery risk [1]. In addition, guidelines recommend lifelong anticoagulation in patients with CTEPH, such as Vitamin K antagonists and Factor Xa inhibitors like rivaroxaban. Reports advise Vitamin K antagonists for those with antiphospholipid syndrome [1,182]. Lastly, while there is benefit for those with CTEPH, anticoagulation treatment use for PAH is unclear. Research suggests that anticoagulation therapies such as warfarin may help improve survival and quality of life in patients with idiopathic PAH, but other studies report no significant advantage in the same group of patients [1,183,184].

Other general therapies include managed exercise training, which improves hemodynamics, oxygen uptake, and quality of life in patients affected by PH [185,186]. ESC/ERC guidelines recommend limiting salt and water consumption to reduce volume overload and hypertension as well as correcting iron deficiency, which often occurs in PH [1]. They also strongly advise women against pregnancy, as studies link pregnancy with higher maternal mortality rates in those affected by PH [1]. ESC/ERC protocol also recommends routine vaccinations to prevent infections such as influenza and pneumococcal pneumonia as a general safety measure [1].

The management of PH patients in Group 2 and Group 3 often focus on addressing the underlying issue with therapies that focus on problems such as heart failure, valvulopathy, or obstructive or restrictive lung diseases. Currently, lung transplants are the only potentially curative treatment for PH associated with chronic lung disease, and clinicians can also consider such transplants for patients in other PH groups who are unresponsive to medications and in a high FC [1].

## 5. Potential Therapies

In addition to FDA-approved medications and general therapies, current investigations are studying potential therapies in pre-clinical or clinical studies. We highlight specific therapies below.

### 5.1. Prostacyclin Analogs

Ralinepag, similar to other FDA-approved prostacyclin analogs listed above, is a potential therapy for PAH. Ralinepag also exhibits similarities mechanistically to other prostacyclin drugs, promoting vasodilation and discouraging smooth muscle cell proliferation through binding to the IP receptor, leading to an intracellular increase in cAMP [187]. In terms of administration, patients can take ralinepag orally [187]. Most pertinently, ralinepag has shown benefits in PAH patients, including improvement in six-minute walk testing and reductions in PVR [187,188]. Currently, clinical studies and data analysis are ongoing via Phase 3 ADVANCE trials, which aim to elucidate the efficacy of ralinepag in endpoints such as time to clinical worsening events and exercise capacity, as well as the drug’s long-term safety and efficacy [189,190,191].

### 5.2. sGC Stimulators

MK-5475 is a sGC stimulator that is in an inhalable dry powder form. Multiple research efforts are currently investigating the drug: for instance, a Phase I study demonstrated that MK-5475 is not only safe but reduces PVR in patients with PH-COPD [192]. MK-5475 has also shown the same benefits in a phase I study which involved patients with PAH [193]. The INSIGNIA-PAH trial, a Phase II and III trial assessing the efficacy and safety of MK-5475 in patients with PAH, was recently finished [194]. Currently, no Phase II trials testing MK-5475 in PH-COPD patients exist.

### 5.3. CGRP

Research finds that reduced CGRP, a potent vasodilator found in cells such as pulmonary neuroendocrine cells, in the plasma of rats affected by hypoxia-induced PH [195,196]. The administration of CGRP can stimulate pulmonary vasodilation in hypoxic-induced PH animal models and attenuate pathological vascular remodeling in PAH rat models in part by suppression of ET-1 release and attenuating the cGMP-AMP synthase (cGAS)-simulator of inferior genes (STING)-nuclear factor kappa-light-chain-enhancer of activated B cells (NFκB) pathway, respectively [197,198,199,200]. CGRP ligands may also activate the calcitonin receptor-like receptor (CLR)/receptor activity-modifying protein 1 (RAMP1) receptor, leading to the triggering of multiple signaling pathways that, in turn, regulate not only intracellular energy metabolic patterns but also reduce the mitochondrial damage involved in PAH rat models [199]. A study also reports that the replacement of the CGRP gene with an adenoviral vector ameliorates hypobaric chamber-induced pulmonary hypertension in mice models [201]. However, researchers would need to perform more studies to investigate CGRP’s efficacy in subjects with PH.

### 5.4. HIF-Related Therapies

Currently, no approved medications exist targeting HIF pathways in PH patients. However, research suggests that inhibitors of the hypoxia-inducible factor 2 (HIF-2) pathway can help improve symptoms such as right heart function and pulmonary remodeling in rat models of PAH and hypoxia-induced PH [202,203]. Moreover, numerous pre-clinical studies have reported similar results with HIF-1 inhibitors [204,205,206,207,208].

### 5.5. BMPR2-Related Therapies

Tacrolimus is an immunosuppressive medication typically used for those with organ transplants or autoimmune diseases. However, tacrolimus also ameliorates right ventricular systolic pressure, right ventricular hypertrophy, and reduced pathological vascular changes in PAH rat models with dysfunctional endothelial BMPR2 signaling [209]. Moreover, tacrolimus potentially rescues BMP signaling in pulmonary artery endothelial cells by blocking an inhibitor protein of BMPR1 and activating SMAD1/5 and mitogen-activated protein kinase (MAPK) signaling [209]. Furthermore, a phase IIa randomized controlled trial showed that PAH patients tolerated tacrolimus well [210]. Still, additional studies need to confirm the efficacy of tacrolimus.

Etanercept targets the BMPR2 pathway in part by inhibiting TNFα, a cytokine that is overexpressed in PAH patients and leads to the downregulation of BMPR2 mRNA in rat PAH models [160,211]. Although research shows that etanercept reduces pulmonary hypertension and lung tissue cytokine levels in rat models of PAH, there are no clinical trials on the drug to date [212].

Studies have also investigated other routes of therapy for in PAH models. For instance, the targeted delivery of exosomes to replace defective BMPR2 has the ability to reverse monocrotaline-induced PH [213]. Studies on rats indicate that SRT2104, an activator of Sirtuin 1 (SIRT1), can mediate the restoration of tuberous sclerosis complex 2 (TSC2), a growth suppressor protein on smooth muscle cells reduced in PAH, to ameliorate disease [214].

### 5.6. Additional Immunological Therapies

Immunosuppressive drugs such as dexamethasone, mycophenolate mofetil, cyclosporine, tacrolimus, and etanercept reduce neutrophil migration, decrease lymphocyte proliferation, and attenuate endothelial cell dysfunction and hemodynamic parameters in animal models of PAH. However, these preclinical findings warrant further translational and clinical investigation [215].

Rituximab, a monoclonal antibody against CD20 (a B-cell protein), has shown modest but not statistically significant symptomatic benefits, as measured by the six-minute walk test in PAH patients [216]. Anakinra, which acts as an IL-1 receptor antagonist, has shown symptomatic benefits in PAH patients, as measured by different heart failure and disease severity scores. However, both will need more robust clinical trials to establish their safety and efficacy [216,217].

### 5.7. Potential Neurostimulation and Autonomic-Related Therapies

There is also increasing evidence for a role for the autonomic nervous system in PH. Studies have suggested both increased sympathetic activity and decreased parasympathetic activity in PAH patients [218,219]. Additional pre-clinical tests suggest a role for imbalanced autonomic activity in other PH subtypes; for instance, both sympathetic denervation and parasympathetic stimulation attenuated pulmonary vascular remodeling in CTEPH rat models [220]. Furthermore, chronic intermittent hypoxia led to rostral ventrolateral medulla (rVLM)-mediated heightened sympathetic tone, although research has not yet directly linked such a finding to PH [221].

#### 5.7.1. Multiple Pharmacological Therapies Have Been Tested to Assess the Role of the Autonomic System in the Pathogenesis of PH

ESC/ERC guidelines, together with other studies, have stated that clinical evidence for beta (β)-blocker use in PH management is currently lacking [1,222]. However, research shows that the beta-blocker carvedilol improves heart rate and right ventricular (RV) function in hypoxia-induced PH rats, inhibits smooth muscle cell proliferation in vitro, and helps control heart rate in patients with PAH [223,224,225]. Moreover, nebivolol, a β1 antagonist and β2 agonist, reduces the proliferation of vasoactive and proinflammatory factors from pulmonary artery endothelial cells from patients with PAH [226]. Other similar drugs, such as bisoprolol and arotinolol, improve RV function by preventing RV hypertrophy and improving RV contractility in rats with monocrotaline-induced PH [227,228]. However, more extensive clinical trials with beta-blockers have not yet been conducted, let alone shown efficacy in patients with PH, although these pre-clinical studies detail some promising prospects [1,222,229,230,231,232,233].

With regard to the parasympathetic nervous system, the acetylcholinesterase inhibitor pyridostigmine attenuates right-sided heart dysfunction and pulmonary remodeling in rat models of hypoxia-induced PH and CTEPH, respectively [219,220].

#### 5.7.2. Non-Pharmacological Therapies: Sympathetic Activity Modulation and Vagal Nerve Stimulation (VNS)

Sympathetic modulation and neurostimulation of parasympathetic nerves could also become promising therapies for PH. For instance, researchers have conducted sympathetic modulation through methods such as sympathetic ganglion block, renal sympathetic denervation, and pulmonary artery denervation (PADN), which itself involves techniques such as radiofrequency ablation or high-energy ultrasound [220,234,235,236]. Studies with animal models of PAH and CTEPH have reported that reducing sympathetic activity attenuates pulmonary vascular remodeling, reduces hemodynamic parameters including right ventricular pressure and mPAP, and ameliorates pulmonary wall thickness [220,237,238,239,240,241,242,243,244]. Mechanistically, sympathetic modulation leads to increased NO signaling, an altered expression of genes that are related to inflammation and vasoconstriction, and the downregulation of the activity of the renin–angiotensin–aldosterone system [220,237,238,239].

Clinically, studies have reported that PADN leads to a decrease in hemodynamic parameters, such as mPAP and PVR, as well as an improvement in exercise capacity and cardiac function in patients with PAH [235,236,245,246]. Other studies suggest that PADN could have similar benefits in those with residual CTEPH [247,248], but more extensive, randomized studies are warranted to assess PADN’s effectiveness in patients with PH [1].

Vagal nerve stimulation (VNS) may also help to ameliorate PH, as suggested by multiple pre-clinical studies. Chronic VNS helps prolong survival, reduce dysautonomia and inflammation, and improve right heart function and hemodynamic parameters in various rat models of PAH [249]. One study also reported that neurostimulation helps to preserve right ventricular function in rats with significant right ventricular overload and PH induced by pulmonary arterial banding [250]. Currently, no clinical studies have investigated the efficacy of VNS in patients with PH.

#### 5.7.3. Potential for Additional Integrative Therapies

Research has not yet investigated manual acupuncture (MA) and electroacupuncture (EA), both of which stimulate somatosensory nerves, for patients with PH. However, EA treatment via specific acupoints ameliorates elevated mPAP, vascular remodeling, and right ventricular hypertrophy in rat models of hypoxia-induced PH, in part by normalizing ET-1 and endothelial nitric oxide synthase (eNOS) imbalances [251]. EA also attenuates pulmonary vascular remodeling, via pathways involving vascular endothelial growth factor (VEGF)/phosphoinositide 3-kinases (PI3K)/Akt and ET-1 reduction, in rats affected by COPD [252]. These findings corroborate those from pre-clinical and clinical studies in different diseases, such as systemic hypertension, asthma, and systemic sclerosis, which report acupuncture’s amelioration of ET-1, NO, and CGRP imbalances [253,254,255,256,257,258,259].

MA and EA have also shown additional benefit in both animal models and patients with comorbidities commonly found in PH, such as heart failure, lung disease, and systemic sclerosis. For instance, studies in patients with COPD have demonstrated acupuncture’s role in improving lung and pulmonary function, exercise capacity and endurance, efficiency of oxygen uptake, oxygen saturation, and quality of life [260,261,262,263,264,265,266]. Additionally, acupuncture improves the ejection fraction and the regulation of pathological ventricular enlargement in subjects with heart failure, both in preclinical and clinical studies [267,268,269]. These findings are listed in Table 2, together with other studies that will be discussed below. Regardless, although the studies do not make a link to PH, it could be possible that acupuncture provides symptomatic benefit to PH patients with these comorbidities. However, more research needs to be conducted, via both pre-clinical and clinical studies, to establish acupuncture as a therapy for comorbidities in those with PH. Moreover, an analysis of seven randomized controlled trials on heart failure patients concluded that acupuncture studies displayed methodological heterogeneity and an inconclusive efficacy [270].

Acupuncture’s mechanisms with regard to these reported benefits have not been fully elucidated. Similar to the PADN studies mentioned above, it is possible that acupuncture mediates its benefit through modulating sympathetic activity: studies have reported that EA can dampen elevated sympathetic responses, including in rat models of cardiac dysfunction such as heart failure and myocardial infarction [269,271,272,273]. In these rats and other sympathetically stressed animals, EA-mediated reduction in sympathetic activity in such heart failure studies depend on C-fibers and thinly myelinated group A delta (Aδ)-fibers within the median nerve [271,272,274,275]. Indeed, such somatosensory input through the median nerve has been shown to activate processes within the hypothalamus, midbrain, and medulla to modulate elevated sympathetic activity, including increasing opioid expression, consequently altering signaling in the rVLM [271,274,276,277] and activating glutamatergic, reciprocal, and excitatory pathways between the arcuate nucleus (ARC) and the midbrain ventrolateral periaqueductal gray (vlPAG) [273,278]. Pre-clinical studies with median nerve stimulation have also suggested EA reduces gamma-aminobutyric acid (GABA) release in the vlPAG, disinhibiting vlPAG neurons, and in turn suppressing sympathetic neuronal activity in the rVLM through a serotonergic-mediated pathway [279,280,281]. EA also activates opioid neurons in the ARC which project to the rVLM, reducing elevated activity of the pre-sympathetic neurons [281]. Lastly, EA can reduce sympathetic activity through other central pathways. For instance, research suggests that the hyperactivity of sympathetic neurons within the hypothalamic paraventricular nucleus (PVN) and their ensuing connections with the rVLM play a role in multiple diseases, including in models of hypoxia-induced PH [282,283,284,285]. Elevated levels of corticotropin-releasing hormone (CRH) synthesis and neuronal activity contribute to this sympathetic overactivity, including in disease models of PH [282,283]. Moreover, studies observe activated CRH neurons in the PVN and nucleus tractus solitarius (NTS) during acute hypoxic conditions leading to increased sympathetic outflow [286,287,288,289,290]. Although not in PH models, studies report that EA reduces CRH signaling in the rVLM and PVN in animal models of stress, cardiovascular disease, and multiple other sympathetic excitatory-related conditions [291,292,293,294]. Reductions in other nitric oxide synthases, including neuronal nitric oxide synthases (nNOS), in the PVN and other areas such as the lung exposed to hypoxia, promote hypoxia-induced PH and are associated with increased sympathetic activity [295,296]. However, these mechanisms with relation to EA effects in PH are not clear, although one study has shown that EA decreases nNOS levels in the hypothalamus in a rat model of systemic hypertension [297].

It is important to note that more studies are necessary not only to establish EA’s efficacy in pre-clinical models of PH (for which there is one study listed, [251]), but also if the EA modulation of sympathetic activity would apply to attenuating symptoms in PH models, and whether the mechanisms above would underlie such a change. There is evidence that heightened sympathetic activity plays a role in PH pathogenesis as mentioned above, but these studies do not yet detail a firm link between acupuncture and PH.

**Table 2:** Studies supporting the role of acupuncture in the pathologies of PH.

References	Model	Technique	Findings Potentially Relevant to Pulmonary Hypertension (PH)
[251]	Pre-clinical Hypoxic-induced PH	Electroacupuncture (EA)	Mean pulmonary arterial pressure (mPAP) ↓, right ventricular (RV) size ↓ Pathological pulmonary remodeling ↓ Serum/lung endothelial nitric oxide synthase (eNOS) ↑, serum/lung endothelin-1 (ET-1) ↓
[253,297]	Pre-clinical Hypertension	EA Non-EA	Sympathetic activity (e.g., via nitric oxide synthase (NOS) pathways) ↓ Serum norepinephrine ↓ Serum interleukins/C-reactive protein ↓ Serum ET-1 ↓, myocardial eNOS ↑
[256,257]	Clinical Hypertension	Non-EA + enhanced external counterpulsation (EECP)	Serum nitric oxide (NO) ↑, serum ET-1 ↓
[260,261,262,263,264,265,266]	Clinical Chronic obstructive pulmonary disease (COPD)	EA Non-EA	Oxygen utilization/efficiency ↑, dyspnea ↓, exercise capacity ↑
[298,299,300,301,302,303,304]	Pre-clinical Systemic inflammation	EA Non-EA	Serum/lung tumor necrosis factor alpha (TNF-α), interleukins ↓ Parasympathetic (vagus) outflow ↑ Ejection fraction ↑
[267,268,269,272]	Pre-clinical Heart failure/cardiac insult	EA	Sympathetic outflow ↓ Heart function ↑ (i.e., left ventricle ejection fraction ↑, left ventricle size ↓
[271,273,274,275,276,277,278,279,280,281,291,292,294]	Pre-clinical Sympathetically stressed	EA	Sympathetic outflow ↓ (i.e., via central opioid, corticotropin-releasing hormone (CRH) pathways) Serum CRH, cortisol, norepinephrine, adrenaline ↓
[305]	Clinical Post-surgery secondary to lung cancer	EA	Arterial oxygen partial pressure (PaO_2_)/fraction of inspired oxygen (FiO_2_) ↑ Superoxide dismutase (SOD) activity ↑ Length of hospital stay ↓
[258]	Clinical Systemic sclerosis	EA	Plasma ET-1 ↓
[306,307,308]	Pre-clinical Lung injury	EA	Lung SOD activity ↑ Serum/lung cytokines ↓ PaO_2_ ↑ Lung injury score ↓
[252,309]	Pre-clinical COPD	EA	Pathological pulmonary remodeling ↓ Lung cytokines ↓ Lung function (i.e., expiratory volume) ↑

PH = pulmonary hypertension; EA = electroacupuncture; ET-1 = endothelin-1, NO = nitric oxide, eNOS = endothelial nitric oxide synthase; EECP = enhanced external counterpulsation; COPD = chronic obstructive pulmonary disease; CRH = corticotropin-releasing hormone; PaO_2_/FiO_2_ = arterial oxygen pressure/fraction of inspired oxygen; SOD = superoxide dismutase. ↓ indicates a decrease in the stated parameter, while ↑ indicates an increase.

**Figure 2:**
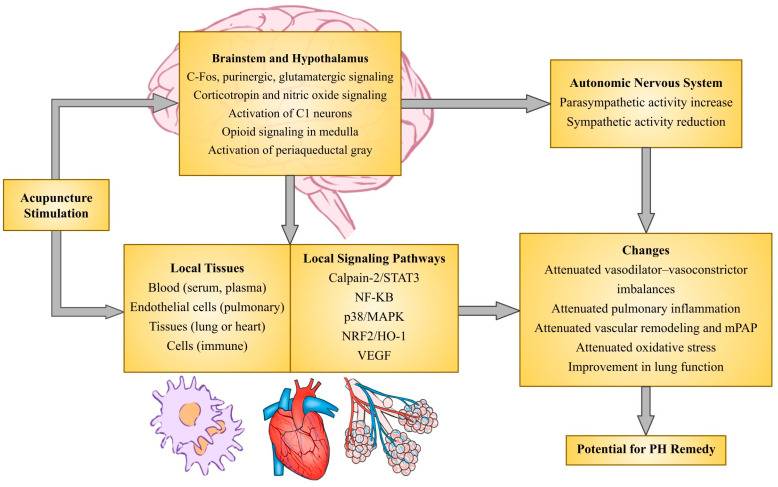
Potential mechanisms of acupuncture-mediated neurostimulation for pulmonary hypertension (PH).

In addition to autonomic dysfunction, acupuncture may ameliorate PH through actions within the lungs. For instance, EA reduces serum levels of the vasoconstrictor ET-1 in patients with systemic sclerosis and systemic hypertension, the former of which is associated with PAH [253,257,258]. As mentioned above, a preclinical study showed EA-mediated reversal of hypoxia-induced PH via the attenuation of elevated mean pulmonary arterial pressure, right ventricular hypertrophy, and pulmonary vascular remodeling and reductions in ET-1 levels [251]. Mounting evidence also shows that neurostimulation techniques such as acupuncture may also reduce inflammation, which is found in various disease pathologies including PH. For instance, multiple studies report that both MA and EA at varying acupoints help reduce serum cytokines—including TNFα, IL-1β, and IL-6—in rat models of endotoxin-mediated inflammation in part through vagal efferents [268,298,299,300,301,302]. Moreover, studies suggest that acupuncture influences the activity of the vagal–adrenal axis through the cholinergic–anti-inflammatory pathway, namely via the activation of the vagus nerve, dopamine release from the adrenal gland, and the suppression of systemic inflammation [268,300,301,310,311]. Lastly, there is evidence suggesting acupuncture’s role in improving pulmonary function and regulating oxidative stress and inflammation. For instance, EA increased lung tissue SOD activity to improve pulmonary lung function in rat models; moreover, EA similarly increased serum SOD activity and improved lung function and recovery in patients recovering from operations due to lung cancer [305,306,312]. Other studies have demonstrated that EA affects multiple signaling pathways to reduce inflammation and oxidative stress in animal models through various other signaling pathways, such as the inhibition of calpain-2 and STAT3 pathways in cardiomyocytes, the activation of local cannabinoid receptors and the inhibition of Toll-like receptor 4 (TLR4)-NF-κB signaling in peripheral immune cells or lung tissue, and the modulation of the nuclear factor erythroid 2-related factor (Nrf2)/heme oxygenase-1 (HO-1) pathway [268,302,303,304,306,307,308]. There is also evidence that acupuncture reduces the expression of genes related to oxidative stress and inflammation as well, limiting ROS and cytokine production, albeit in animal models with varying conditions like inflammation and ischemia-induced hypoxia [303,313,314]. Other studies indicate that acupuncture could also mediate immune cell migration in lung tissue [309,315]. Figure 2 below diagrams the mechanisms this section details.

Acupuncture helps manage multiple other diseases. Clinically, care teams use acupuncture to help manage various types of pain, especially chronic pain [316]; however, there are also studies suggesting that acupuncture could be beneficial in managing symptoms in diabetes [317,318], depression and sleep disorders [319,320], hypertension, cardiovascular problems, and other conditions listed above. A considerable number of the studies listed are pre-clinical; hence, more investigations are needed to establish acupuncture’s efficacy in patients affected by PH, given acupuncture’s potential role in mediating pulmonary vascular remodeling, autonomic dysfunction, and inflammation, as mentioned in studies above.

## 6. Conclusions

PH is a complex disease that affects millions of people globally. While studies have revealed multiple pharmacological treatments alleviating suffering and mechanisms associated with the disease, furthser investigations would be beneficial in exploring both emerging therapies and complementary non-pharmacological treatments. Moreover, given that PH is on the rise in part due to the greater utilization of diagnostics [26,27], more studies are necessary for care teams to offer preventive and potentially curative treatments for PH.

## Figures and Tables

**Table 1:** Etiological and clinical classifications of pulmonary hypertension (PH) [1].

Group 1: Pulmonary Arterial Hypertension (PAH) 1.1: Idiopathic 1.2: Heritable 1.3: Associated with drugs and/or toxins 1.4: Associated with diseases such as connective tissue disease and human immunodeficiency virus (HIV) infection 1.5: PAH with features of venous/capillary (PVOD/PCH) involvement 1.6: Persistent PH of the newborn Group 2: PH associated with left heart disease (PH-LHD) 2.1 Heart failure 2.2 Valvular heart disease 2.3 Congenital/acquired cardiovascular conditions leading to post-capillary PH Group 3: PH associated with lung diseases and/or hypoxia 3.1 Obstructive lung diseases 3.2 Restrictive lung diseases 3.3 Mixed obstructive/restrictive lung diseases 3.4 Hypoventilation syndromes 3.5 Hypoxia without lung disease (e.g., staying at a high altitude) 3.6 Developmental lung disorders Group 4: PH associated with pulmonary artery obstructions 4.1 Chronic thrombo-embolic PH (CTEPH) 4.2 Other pulmonary artery obstructions (e.g., sarcomas, tumors, arteritis without connective tissue disease, etc.) Group 5: PH with unclear or multifactorial mechanisms

Reproduced with permission of the © European Society of Cardiology and the European Respiratory Society 2024: European Respiratory Journal 61 (1) 2200879; DOI: 10.1183/13993003.00879-2022. Published 6 January 2023 [1]. HIV = human immunodeficiency virus; PCH = pulmonary capillary hemangiomatosis; PVOD = pulmonary veno-occlusive disease.
